# Outcome of patients with advanced solitary fibrous tumors: the Centre Léon Bérard experience

**DOI:** 10.1186/1471-2407-13-109

**Published:** 2013-03-11

**Authors:** Alice Levard, Olfa Derbel, Pierre Méeus, Dominique Ranchère, Isabelle Ray-Coquard, Jean-Yves Blay, Philippe A Cassier

**Affiliations:** 1 Department of Medical Oncology, Centre Léon Bérard, 28 rue Laennec, 69008, Lyon, France; 2 Department of Surgical Oncology, Centre Léon Bérard, 28 rue Laennec, 69008, Lyon, France; 3 Department of Anatomopathology, Centre Léon Bérard, 28 rue Laennec, 69008, Lyon, France; 4 Department of Medical Oncology, Centre Léon Bérard, Lyon, France

## Abstract

**Background:**

Solitary Fibrous Tumor is a rare type of soft tissue tumor of intermediate malignant potential which may recur or metastasize in 15-20% of cases. Data on the management of patients with advanced SFT is scarce: chemotherapy has been described as ineffective, while recent data suggests that anti-angiogenic therapies may be more efficient.

**Methods:**

We conducted a retrospective study on patients treated for advanced SFT at a single institution: from January 1994 to December 2011, 30 patients were treated in the Centre Léon Bérard for an advanced SFT.

**Results:**

Twenty-three patients received cytotoxic chemotherapy as first-line therapy. Best responses were 2 (9%) partial responses, 13 (57%) stable diseases (SD) and 8 (35%) progressive diseases (PD). Median Progression Free Survival (PFS) was 5.2 (95% CI: 3.2-7.1) months and 9 patients were free of progression at 6 months. Ten patients received an anti-angiogenic treatment (sunitinib or pazopanib) as a 2^nd^, 3^rd^ or 4^th^ line. Best responses were 5 SD and 5 PD; median PFS was 5.1 months (95% CI 0.7-9.6). Four patients (36%) were progression-free for more than 6 months. Two patients receiving pazopanib were without progression at 6 and 8 months and two patients receiving sunitinib were free of progression at 30 months.

**Conclusion:**

Response rate with standard chemotherapy was low and PFS appear similar between cytotoxic chemotherapy and anti-angiogenic agents.

## Background

Solitary fibrous tumor (SFT) is a rare type of soft tissue tumor that was during many decades, assimilated to hemangiopericytoma (HPC). However, with time it became clear that the diagnostic criteria for hemangiopericytoma were too loose to adequately reflect reality. Therefore, the last World Health Organization classification published in 2006 identifies SFT as a distinctive entity. While true HPC still exists, its definition is now more specific and delineated [1,2]. Finally, sarcomas with hemangiopericytic features are separated from HPC and SFT. Initially described as a mesothelial pleural lesion, SFT is now recognized to arise in ubiquitous anatomical sites and may occur both in mesothelial tissues (pleura, peritoneum, pericardium) and soft tissues or visceral organs (lung, meninges, thigh, thyroid, etc.,) [3]. It generally follows a benign clinical course. Nevertheless, according to Park and Araujo [4], it may recur either locally or at distant sites in 15 to 20% of the patients. Although criteria were defined to help clinicians differentiate malignant lesions from more benign ones, predicting the clinical course of these rare lesions remains challenging. In most cases, malignant lesion are characterized by large tumor size, high mitotic index (more than 4 mitoses per 10 high-power fields), nuclear pleomorphism, high cellularity and the presence of necrosis and/or hemorrhage [5]. However, the relationship between histological features and clinical behavior of SFT is not so clear and these tumors still have unpredictable course.

When SFT is localized, one of the most important prognostic factor is the quality of the initial excision, with free margins [6]. Thus, the ten-year overall survival rate varies between 54 and 89% between series [7,8]. When the tumor cannot be removed surgically or when metastases occur, chemotherapy and/or radiotherapy can be proposed as palliative treatments. Data on the role of chemotherapy in the treatment of patients with advanced SFT is currently limited to small retrospective studies. In most of these studies, the drugs are those used for treatment of soft tissue sarcomas (STS) such as anthracyclines with or without ifosfamide [9,10], trabectedin [11], and gemcitabine combined with docetaxel. More recently, several case-reports and small series have suggested that anti-angiogenic drugs may have activity in SFT. Park et al. reported in 2009 their experience on 14 patients treated with bevacizumab combined with temozolomide. Based on Choi criteria [12], 11 patients had a partial response (PR), 2 had stable disease (SD) and 1 had progressive disease (PD) as their best response. However only 2 patients in this study achieved a PR based on RECIST [13]. Case-reports of patients achieving long term tumor control with sunitinib, sorafenib [14], or imatinib [15] have been published. In the present study we sought to assess the outcome of patients with advanced inoperable SFT managed at our institution with standard chemotherapy and anti-angiogenic agents.

## Methods

This retrospective study was approved by the ethics committee CPP Lyon Est IV. The Centre Léon Bérard (CLB) sarcoma database was searched for patients with a diagnosis of SFT. Two hundred and thirty three patients’ files were identified between January 1994 and December 2011. The vast majority of these patients were registered in the CLB database for histology review or multidisciplinary meeting discussion but only 66 patients were actually managed at the CLB, of which 30 had advanced disease and are the subject of this report.

Data were extracted from individual patients’ files and analysed. All cases were reviewed by an expert pathologist in the field of sarcomas (DR). Patients and tumor characteristics were described using the median and range for continuous variables and percentages with 95% Confidence Interval (95% CI) for categorical variables. Response was assessed using RECIST 1.0 [16] and described as a response rate (RR) defined as the percentage of patients with PR or complete response (CR). Overall survival (OS) was defined as the time from the date of diagnosis of advanced disease to the date of death from any cause. Progression-free survival (PFS) was calculated from the date a systemic treatment was started to the date of disease progression or death of any cause, whichever occurred first. Survival times were plotted using the Kaplan-Meier method and compared using the log-rank test. P-values of 0.05 or less were considered statistically significant.

## Results

### Patients’ characteristics

Thirty patients were identified as having advanced disease of which 18 were males. The median age at initial diagnosis was 57.7 (range 24.7-83.3) years, while age at diagnosis of advanced inoperable disease was 62.3 (range 34.7-87.2) years. The primary tumor was localised in pleura (n=14, 47%), pelvis (n=4, 13%), meninges or cerebellum (n=3, 10%), limb (n=3, 10%), visceral organs (sigmoid and bladder, n=2, 7%), spine (n=2, 7%), peritoneum (n=1, 3%) and mediastinum (n=1, 3%). Twenty five patients underwent surgical resection of their primary tumor, while five (17%) had unresectable disease at presentation. Seventeen patients had metastatic disease and the most common sites of metastasis were the lung (n=9, 53%), pleura (n=5, 29%) or peritoneum (n=3, 18%) but liver, bone and lymph node metastases were also noted. Of these 17 patients, 5 had metastases at the time of presentation while 12 developed metastasis during follow-up (median time from initial diagnosis 47.9 months; range 12.0 – 215.6 months). The main patient characteristics are summarized in Table 1.

**Table 1:** Patient characteristics’ for the whole cohort

**Patients**		**N**
Sexe		
	Men	18
	Women	12
Age, median (range) (yrs		
	At initial diagnosis	57.7 (24.7 – 83.3)
	At advanced inoperable disease	62.3 (34.7 – 87.2)
		
Primary tumor site		
	Pleura	14
	Meninges/Cerebellum	4
	Pelvic	4
	Limbs	3
	Visceral organs	2
	Peritoneum	1
	Mediastinum	1
	Spine	1
Metastatic disease		
	Yes	17
	No	13
Metastatic sites		
	Lung	9
	Pleura	5
	Peritoneum	3
	Liver	2
	Bone	2
	Lymph nodes	2
Primary surgery		
	Yes	25
	No	5
Doege-Potter Syndrome		2

**Figure 1:**
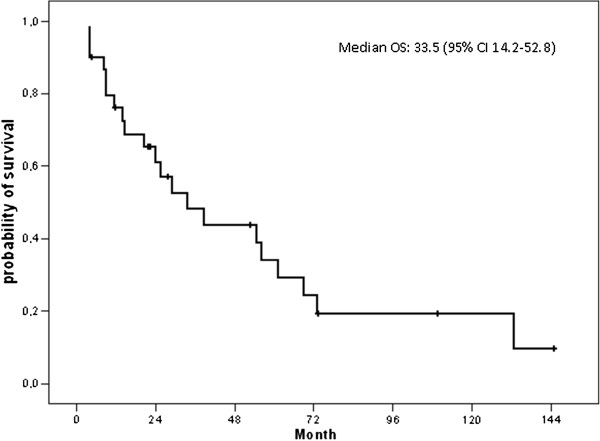
Overall survival for the whole cohort (n=30).

For 13 patients the initial diagnosis was not that of SFT and for most of these patients the diagnosis of SFT was made at relapse. In these cases, the following diagnoses were initially raised: fibroma (n=3, 23%), gastro-intestinal stromal tumor (GIST, n=2), mesothelioma (n=2, 15%), fibrosarcoma (n=1, 8%), leiomyosarcoma (n=1, 8%), angiosarcoma (n=1, 8%), histiofibrocytoma (n=1, 8%), hamartochondroma (n=1, 8%), and meningioma (n=1, 8%). After a median follow-up of 109 months for surviving patients, 21 patients had died, 19 from progression of SFT, one from a treatment related complication (thrombotic event during anti-angiogenic therapy) and one from unknown cause, and 3 patients were lost to follow-up at 4.6, 11.8 and 144.7 months of follow-up. The median overall survival for the whole cohort (n=30) was 33.5 months (95% CI 14.2-52.8) from the date of advanced inoperable disease (Figure 1).

### First line treatment

Of the 30 patients, 3 received radiotherapy only, 2 patients benefitted from surgery, 2 patients from imatinib and 23 received chemotherapy as first-line treatment.

Three patients received radiotherapy as their sole treatment modality, in most cases for locally advanced disease without metastases. The first patient was a 55-year old lady with a skull-base SFT who had a partial response after radiotherapy and was still alive and progression-free after 70 months of follow-up. The second patient was a 67-year old gentleman with a skull-base SFT who experienced disease progression shortly (2 months) after the completion of therapy, did not received further therapy and was lost to follow-up 4 months after the completion of radiotherapy. The third patient was an 81-year old gentleman who had a recurring pleural SFT (after 2 operations), had stable disease after radiotherapy but had disease progression 19 months after the completion of radiotherapy and died shortly after (22 months). Two patients who had R2 resection of pleural SFT and were deemed not re-operable were offered pseudo-adjuvant chemotherapy. Both had disease progression 5 and 26 months after the completion of chemotherapy and were then offered pazopanib. Two patients who were initially diagnosed as GISTs received imatinib as first line treatment; both had disease-progression after 3 months of treatment. One of these patients underwent surgical excision of his disease because of symptomatic uncontrolled hypoglycaemia and was lost to follow-up shortly after; pathological analysis of the surgical samples allowed correcting the diagnosis to that of SFT. Because of the poor response to imatinib, the second patient underwent a percutaneous, ultrasound-guided biopsy of his pelvic mass, on which the diagnosis of SFT was done (and no KIT or PDGFRA mutation was found). This patient received sunitinib as second-line therapy and had stable disease for 30 months.

### Response to first line cytotoxic chemotherapy

Twenty-three patients received cytotoxic chemotherapy as their first-line of treatment for advanced measurable disease (therefore excluding the 2 patients who received pseudo-adjuvant chemotherapy): 14 were male and 9 were female. Their median age at the time of treatment start was 65 (range 37–86) years old. Main characteristics of patients who benefited from first-line cytotoxic chemotherapy are indicated in Table 2. First line consisted in a doxorubicin-based regimen for 19 patients that were treated with either doxorubicin alone (n=9), pegylated liposomal doxorubicin (n=1) or a doxorubicin based combination (with ifosfamide n=8 or palifosfamide n=1). The 4 other patients received vinorelbine (n=1), paclitaxel (n=1), carboplatin and paclitaxel (n=1) and brostallicin (n=1).

**Table 2:** Characteristics of patients who received first-line cytotoxic chemotherapy

**Patient**	**Primary site**	**First surgery**	**Delay 1**^**st **^**diagnosis / advanced disease (months)**	**Metastatic/Locally advanced**	**Treatment for advanced disease before systemic treatment**	**First-line treatment**	**PFS/OS** **(months)**
**1**	Pleura	R1	150	Metastatic	Surgery (3)	AI	6,9 / 22,2
**2**	Peritoneum	R0	100	Metastatic	No	AI	14,5 / 70,8
**3**	Pleura	R1	12	Metastatic	No	AI	5,2 / 29
**4**	Tip	N	0	Locally advanced	No	PLD	1,7 / 6,4
**5**	Pleura	R1	20	Metastatic	No	Adriamycine	1,4 / 22,4
**6**	Cerebellum	Unk	128	Metastatic	Surgery (1)	Brostallicin	4,8 / 34,5
**7**	Pelvic	N	0	Metastatic	Radiotherapy	Adriamycine	9,3 / 32,8
**8**	Pleura	R0	20	Metastatic	Surgery (1)	AI	2,4 / 21,2
**9**	Mediastinum	N	0	Locally advanced	No	MAID	9,1 / 12,4
**10**	Pleura	Unk	52	Locally advanced	Surgery (1)	Adriamycine	60 / 68,8
**11**	Shoulder	R1	16	Locally advanced	No	AI	9 / 19,4
**12**	Pelvic	R0	0	Metastatic	No	Adriamycine	1,5 / 5,7
**13**	Pleura	R2	200	Locally advanced	Surgery (1)	Adriamycine-Palifosfamide	6,7 – 10,7
**14**	Pelvic	R0	156	Locally advanced	No	Adriamycine	17,5 / 50,8
**15**	Pleura	R0	11	Metastatic	No	Adriamycine	2,1 / 2,7
**16**	Spine	Unk	96	Locally advanced	Surgery (1)	AI	5,9 / 60
**17**	Tip	N	0	Metastatic	No	Adriamycine	4 / 4
**18**	Spine	R2	120	Metastatic	Surgery (1) and RT	AI	2,9 / 32,3
**19**	Pleura	R1	216	Metastatic	Surgery (1) and RT	Adriamycine	12,5 / 27,3
**20**	Pleura	R1	12	Locally advanced	No	Carboplatin-Paclitaxel	1,0 / 2,1
**21**	Bladder	R0	32	Metastatic	No	Brostallicin	1,8 / 1,9
**22**	Pleura	R0	27	Metastatic	No	Paclitaxel	0,4 / 0,4
**23**	Pleura	Unk	64	Metastatic	Surgery (2)	Vinorelbin	5,2 / 64,5

AI: Adriamycin-Ifosfamide; PLD: Pegylated Liposomal Doxorubicin; Unk: Unknown; RT: Radiotherapy; MAID: mesna, adriamycin, ifosfamide and dacarbazine; Surgery (number of surgeries).

Overall, only 2 PR were observed (RR = 9%), both in patients receiving doxorubicin-based chemotherapy (1 treated with single agent doxorubicin and 1 treated with both doxorubicin and ifosfamide). Thirteen patients (57%) had SD while 8 (35%) had PD as their best response and nine patients (39%) were free of progression at 6 months.

**Figure 2:**
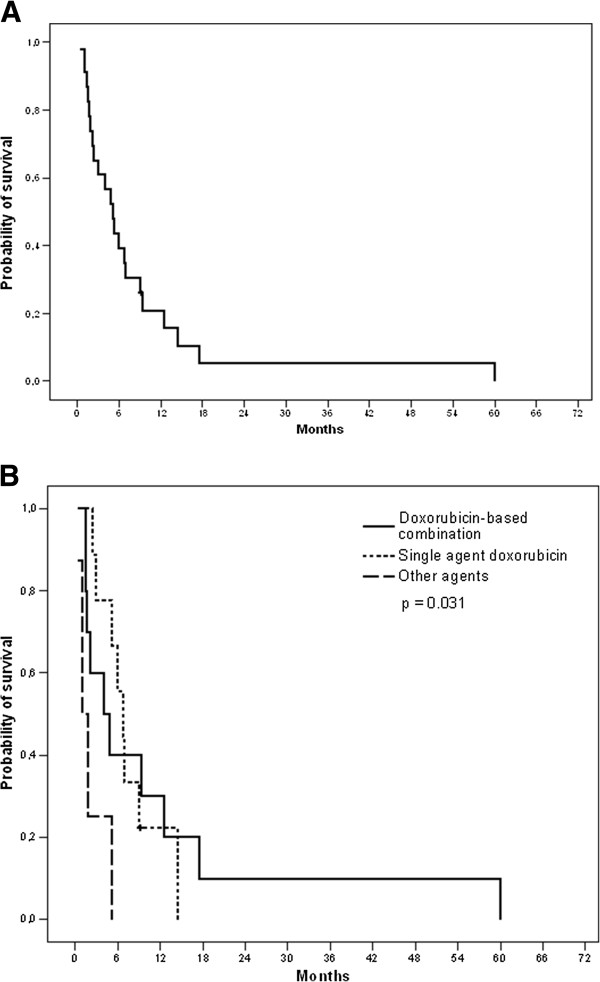
Progression-Free Survival for patients receiving first-line chemotherapy (n=23) (Panel A) and according to the type of chemotherapy (Panel B).

The median PFS was 5.2 (95% CI 3.2-7.1) months and improved when both doxorubicin and ifosfamide were combined (6.7 months) compared to single agent doxorubicin (4.0 months) or other agents (1.0 month), (p = 0.031) (Figure 2).

Using cytotoxic chemotherapy, the main adverse effects identified were grade 4 neutropenia (doxorubicin alone, n=2; doxorubicin and ifosfamide, n=2), grade 4 thrombopenia (doxorubicin and ifosfamide, n=2), grade 3 anemia (doxorubicin and ifosfamide, n=1), pulmonary embolism (1 patient treated with doxorubicin and ifosfamide) and one patient had doxorubicin-induced grade 3 congestive heart failure.

### Other lines of therapy

Twenty one patients received second line therapy, in 12 patients second line treatment was cytotoxic therapy: trabectedin (n=5), gemcitabine (n=3), doxorubicin (n=1), brostallicin (n=1), ifosfamide (n=1) and cisplatin (n=1). The 9 other patients received targeted therapies consisting of imatinib (n=1), pazopanib (n=4), sunitinib (n=2) or other investigational agents (n=2). Follow-up information was not available for one patient and median PFS was 3.4 months (95% CI: 2.2-4.7).

Fourteen patients received third line therapy: gemcitabine (n= 4), trabectedin (n=3), vinorelbine (n=1), pazopanib (n=2), doxorubicin (n=1), etoposide (n=1), cisplatin (n=1) and other investigational agents (n=2). Their median PFS was 4.3 months (95% CI: 1.7-6.8).

Six patients received a fourth line of systemic therapy: sunitinib (n=2), gemcitabine-docetaxel combination (n=2), trabectedin (n=1) and weekly doxorubicin (n=1). Three patients received fifth line therapy: trabectedin (n=1), weekly paclitaxel (n=1) and metronomic oral cyclophosphamide (n=1).

### Response to anti-angiogenic treatment

**Figure 3:**
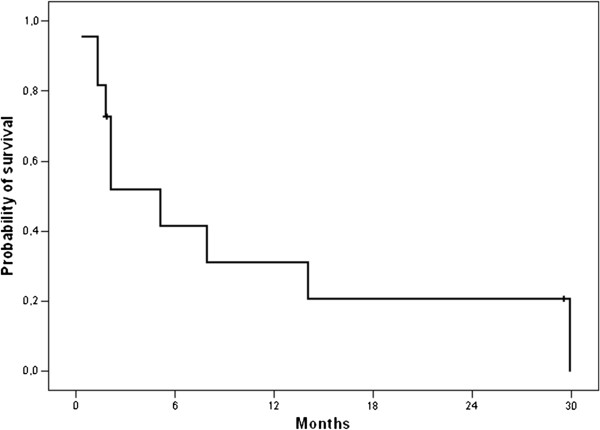
Progression-Free Survival for patients treated with anti-angiogenic agents (n=10).

Ten patients received an anti-angiogenic drug (Table 3): 6 received pazopanib (800 mg once daily) while 4 received sunitinib (37.5 mg once daily). These oral TKI were administered as second line in most patients (n=6) while 4 patients received an anti-angiogenic TKI as 3^rd^ and 4^th^ lines (2 patients each). There were no objective response observed and 5 patients had SD as their best response (3 of 4 treated with sunitinib and 2 of 6 treated with pazopanib). The median PFS on anti-angiogenic was 5.1 months (95% CI 0.0-13.4) (Figure 3) and 4 patients (40%) were progression-free for more than 6 months at 8.0 and 14.0 (pazopanib), and 29.5 and 29.9 months (sunitinib).

**Table 3:** Anti-angiogenic treatments

**Patient**	**Treatment**	**Line**	**Best response**	**PFS (months)**	**OS (months)**
**1**	Sunitinib	4	SD	30	34
**2**	Pazopanib	3	SD	8	15
**3**	Pazopanib	2	PD	2	4
**4**	Sunitinib	4	PD	2	5
**5**	Pazopanib	2	SD	14	19
**6**	Pazopanib	2	PD	0,3	0,3
**7**	Pazopanib	3	PD	2	5
**8**	Sunitinib	2	SD	2	33
**9**	Sunitinib	2	SD	30	50
**10**	Pazopanib	2	PD	4	34

One patient died because of a thrombotic event during the first month of treatment with pazopanib. Elevated liver enzymes (ALT and/or AST) grade 3, anorexia grade 3 and anemia grade 3 were described in three patients receiving pazopanib (one each). Grade 3 sunitinib-induced diarrhea and heart failure were seen in the same patient resulting in discontinuation of the agent.

## Discussion

SFT are rare tumors, especially in the advanced phase as only a minority of patients will eventually recur after primary surgical management. Therefore data regarding the actual effectiveness of systemic therapy in this setting is limited and stems from small retrospective studies. Our aim in this study was to gain further knowledge on patients with SFT managed at our institution and add to the current body of literature on the subject. As anti-angiogenic therapy appears promising in the management of patients with advanced SFT and standard chemotherapy was reported to have limited efficacy we chose to describe both of these approaches separately.

As first line therapy, most patients received a doxorubicin-based regimen similar to patients with soft tissue sarcomas (STS). Indeed, despite an important and well known heterogeneity, most STS subgroups are managed similarly, thus data are extrapolated to SFT and doxorubicin-based regimen appears as a standard in the first-line setting. However, the response rate in our series is low but appears comparable to previously reported data [9]. As previously noted, toxicity is not negligible. Combination therapy may increase response rate but has not been showed to increase OS in patients with STS. Several patients in our series received subsequent lines of chemotherapy, in most cases trabectedin and gemcitabine. In this setting, some patients derived some benefit in terms of tumor control as demonstrated by the median PFS, although no RECIST-defined response was seen.

However, because the efficacy of standard chemotherapy is limited in STS, much hope has grown around the development of targeted therapies. In line with this, patients receiving anti-angiogenic agents such as pazopanib and sunitinib derived some benefit from therapy. Although no partial response was noted, tumor control appeared similar to that of first line chemotherapy (PFS = 5.1 months versus 5.2 months) and appeared longer than that seen with second or third-line chemotherapy, although no statistical comparison was made. Furthermore, several patients achieved long term stable disease including 3 patients which remained on anti-angiogenic therapy for more than a year. These results are comparable to those reported by Stacchiotti et al. [17], who treated 35 patients with advanced SFT most of whom were pre-treated with chemotherapy (25 of 35) with sunitinib 37.5 mg daily. The median duration of the treatment was 5 months (1 week-27 months). Response rate with RECIST assessment was 6.5% (2 of 31 assessable patients had a PR). Seventeen patients had SD (54%) and 12 had PD (39.5%) as their best response. The median PFS was 6 months (CI 95% 4.03-8.01) and median OS was 16 months (CI 95% 12.07- 25.9). One patient had a response that lasted 22 months. In another phase II study evaluating sunitinib in 48 patients with non-GIST sarcomas. In another phase II study evaluating sunitinib in 48 patients with non-GIST sarcomas the overall RECIST response rate was 2%. Three of these 48 patients had SFT. No partial or complete response was reported but long-lasting stable disease were observed for 24 and 58 weeks in 2 patients [18,19]. Another case-report described a 4-months stable disease with sunitinib 50mg daily for a woman with peritoneal progressive disease. Treatment was discontinued because of toxicity but disease control was maintained for more than 6 months after the end of the treatment [14]. In a recently reported phase II study conducted by the French Sarcoma Group 5 patients with malignant SFT were treated with sorafenib: none had a PR or CR [20].

Overall our data underline the modest activity of standard chemotherapy in SFT, nevertheless PFS and RR do not appear significantly lower that what is commonly observed in other STS subtypes. Furthermore, although anti-angiogenic agents have interesting activity in SFT our data and those reported by others suggest that this subtype is only modestly more sensitive than other subtypes of sarcoma. This however would need to be assessed in a prospective trial which is greatly needed in these rare tumors.

## Competing interest

The authors declare that they have no competing interest. This study was not funded.

## Authors’ contributions

AL and PAC designed the study, collected and analyzed the data and wrote the manuscript. AL, PAC, OD, PM, IRC and JYB provided study participants. DR reviewed the pathology samples. All authors reviewed and approved the final manuscript.

## Pre-publication history

The pre-publication history for this paper can be accessed here:

http://www.biomedcentral.com/1471-2407/13/109/prepub
